# Ezh2 is not required for cardiac regeneration in neonatal mice

**DOI:** 10.1371/journal.pone.0192238

**Published:** 2018-02-21

**Authors:** Abdalla Ahmed, Tao Wang, Paul Delgado-Olguin

**Affiliations:** 1 Translational Medicine, The Hospital for Sick Children, Toronto, ON, Canada; 2 Department of Molecular Genetics, University of Toronto, Toronto, ON, Canada; 3 Human Biology Program, University of Toronto, Toronto, Ontario, Canada; 4 Heart & Stroke Richard Lewar Centre of Excellence, Toronto, ON, Canada; Murdoch Childrens Research Institute, AUSTRALIA

## Abstract

The neonatal mouse heart has the remarkable capacity to regenerate lost myocardium within the first week of life. Neonatal cardiomyocytes re-express fetal genes that control cell proliferation after injury to promote regeneration. The loss of regenerative capacity of the heart one week after birth coincides with repression of a fetal transcriptional program coordinated by epigenetic regulators. The histone methyltransferase enhancer of zeste homolog 2 (Ezh2) is a repressor of fetal cardiac transcriptional programs and suppresses cardiomyocyte cell proliferation, suggesting a potential function in heart regeneration. However, it was recently demonstrated that Ezh2 is dispensable for heart regeneration in the neonatal heart. Here, we provide evidence supporting this finding and demonstrate that Ezh2 deficiency does not affect regeneration of the neonatal heart. We inactivated Ezh2 in differentiating embryonic cardiomyocytes, which led to depletion of histone H3 trimethylated at lysine 27 (H3K27me3). *Ezh2* deficiency in cardiomyocytes did not affect clearance of the fibrotic scar in myocardial infarction (MI) and apical resection models of cardiac injury at post-natal day 1 (P1). Similarly, cardiomyocyte-specific loss of Ezh2 did not affect fibrotic scar size after MI or apical resection at P7, suggesting that it does not extend the regenerative time window. Our results demonstrate that *Ezh2* is not required for innate neonatal cardiac regeneration.

## Introduction

The adult mammalian heart has limited capacity for regeneration and repair, owing partially to a progressive reduction in cardiomyocyte (CM) proliferative capacity after birth [1–3]. Minimal CM proliferation in the adult heart leads to limited cardiac muscle regeneration and increased fibrotic scarring in response to cardiac injury, thus impairing heart function and promoting progression towards heart failure [4]. Notably, neonatal mice can regenerate their hearts after myocardial infarction (MI) induced by ligation of the coronary artery, or by resection of the heart apex [5, 6]. During the first week after injury, the lost myocardium is replaced by fibrotic tissue scar, which is completely resolved three weeks post-injury [5]. In response to cardiac injury induced one day after birth (P1), resident CMs revert to a more immature, proliferative state to replenish dead cardiomyocytes [7]. This process involves global changes in gene expression affecting >500 genes, with genes that activate cell proliferation being up regulated, while genes encoding structural proteins are down-regulated [7]. The mouse cardiac regenerative capacity is lost one week after birth [5], as CMs acquire a more stable transcriptional program and are no longer able to re-activate the fetal gene program in response to cardiac injury induced seven days after birth (P7) [7]. The mechanisms preventing re-activation of fetal regenerative gene programs in the mature heart remain elusive.

Repressive histone methylation stabilizes chromatin configurations and maintains transcriptional repression in embryonic cardiac progenitor cells and their differentiated descendants [8, 9]. The histone methyltransferase enhancer of zeste homolog 2 (Ezh2), the catalytic component of the polycomb repressive complex 2 (PRC2), represses embryonic cardiac transcriptional networks by depositing H3K27me3, and is required for cardiovascular development [9–12]. Ezh2 silences fetal cardiac genes by promoting a repressive chromatin environment at its targets, preventing aberrant activation of embryonic gene programs in adulthood, thus maintaining cardiac homeostasis [10]. Moreover, it has been shown that downregulation of Ezh2 by targeting its upstream regulator miR-26a, causes increased proliferation of cultured mouse CMs, suggesting that Ezh2 might repress proliferative gene programs [13]. Incapacity of the mature heart to re-activate genes associated with cell proliferation underlies loss of cardiac regeneration capacity; however, how epigenetic regulators control the regenerative response is still poorly understood.

Here, we investigated the requirement of Ezh2 in the regenerative response in the neonatal mouse heart. We show that inactivation of Ezh2 and decreasing the global levels of H3K27me3 in CMs does not affect the regenerative response of the neonatal heart after myocardial infarction or apical resection.

## Materials and methods

### Ethics statement

Mice procedures agreed with Canadian Council for Animal Care guidelines, and were approved by the Animal Care Committee at The Centre for Phenogenomics, Animal Use Protocol 17-0236H.

### Mice and cardiac injury

Mice were housed under a 12:12-hour light-dark cycle and allowed standard laboratory chow and tap water *ad libitum*. The following mouse strains were used: *Ezh2*^*fl/fl*^ [14], and *Myh6-Cre* [15]. Neonatal MI and apical resection were performed as previously described [16]. Briefly, P1 or P7 pups were randomized and anaesthetized by hypothermia for 5 min prior to thoracotomy. To induce MI, the left anterior descending (LAD) coronary artery was ligated with a 6–0 non-absorbable polypropylene suture (Medtronic, Minneapolis, MN, USA). For apical resection, the apex of the heart was resected using fine scissors. Mice were allowed to recover under a heat lamp before being returned to their mother. Mice were sacrificed 1 or 3 weeks after surgery by decapitation according to approved protocols and hearts were collected for analysis.

### Gene expression analysis by qPCR

Total RNA was isolated from whole ventricles using Trizol LS Reagent (Invitrogen), according to manufacturer’s guidelines. 1 μg of total RNA was used to synthesize cDNA using the qScript cDNA SuperMix kit (Quantabio). qPCR reactions were prepared using SensiFAST SYBR No-ROX Kit (Bioline), and ran on a CFX384 Touch Real-Time PCR Detection System (Bio-Rad) using the following primers: Ezh2 (Forward: 5’-ACCACAGGATAGGCATCTTTG-3’; Reverse: 5’-GGGAAGAGGTAGTAGATGTCAAG-3’), and Rpl13a (Forward: 5’-TCCCTCCACCCTATGACAAG-3’; Reverse: 5’-GTCACTGCCTGGTACTTCC-3’).

### Histology and immunofluorescence

Hearts were fixed in 4% PFA overnight at 4°C, dehydrated in an ethanol series, and embedded in paraffin. Serial 5 μm cross-sections were obtained from paraffin-embedded hearts. Sections were rehydrated by reversal of the ethanol series prior to processing by Masson's trichrome staining. Fibrotic scar area post-MI was quantified using ImageJ software (National Institutes of Health, Bethesda, Maryland, USA) as a percentage of the total left ventricle area. Sections were immunostained as previously described [17]. The primary antibodies and dilutions used were as follows: H3K27me3 (Cell Signaling, 9733, 1/1000), pHH3 (Millipore, 09–797, 1/30000), Ki67 (Millipore, AB9260, 1/100), and α-actinin (Sigma, A7811, 0.13/200).

Sections were imaged using a Nikon Eclipse Ni-U microscope. Images were processed using NIS-Elements (Nikon Instruments Inc., Melville, New York, USA) and ImageJ software. Immunofluorescence images were obtained using an Olympus IX81 Quorum spinning disk confocal microscope (Quorum Technologies Inc.).

### Statistics

All values are expressed as mean ± SE. Data from quantifications was analyzed by unpaired Student t-test. Significance was considered when *p* < 0.05.

## Results

### Ezh2-deficient cardiomyocytes have reduced levels of H3K27me3

**Fig 1:**
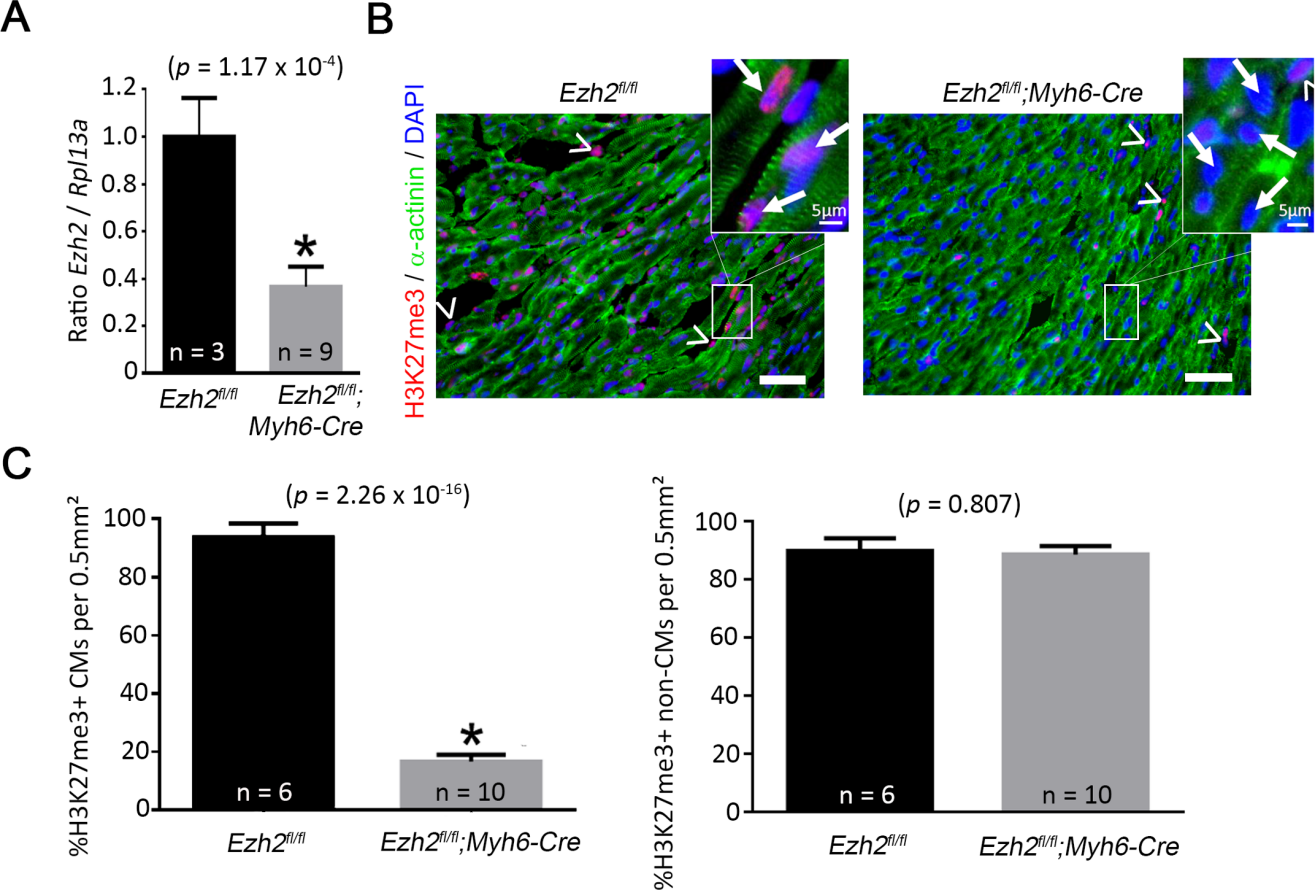
*Ezh2*-deficient cardiomyocytes have reduced levels of H3K27me3. (A) Relative expression of *Ezh2* mRNA in whole ventricles of control (*Ezh2*^*fl/fl*^) and mutant (*Ezh2*^*fl/fl*^*;Myh6-Cre*) mice. (B) Immunofluorescence of H3K27me3 (red) in sections of hearts from P14 control and mutant mice. Cardiomyocytes (CMs) are evidenced by α-actinin (green), nuclei were counterstained with DAPI (blue). Arrowheads point to non-cardiomyocytes. Arrows point to cardiomyocytes. (C) Quantification of H3K27me3-positive (+) CMs and non-CMs. Scale bar = 50 μm. Bars represent the mean +/- SEM.

Inactivation of Ezh2 in a subpopulation of anterior heart field cardiac progenitor cells results in failure to down-regulate fetal gene programs, leading to cardiac hypertrophy during adulthood [10]. However, the function of Ezh2 in the regenerative response of the neonatal heart is poorly understood. To determine the requirement of Ezh2 in heart regeneration, we inactivated *Ezh2* in differentiating CMs. We crossed mice carrying *LoxP* sites flanking exons encoding the catalytic domain of *Ezh2* with transgenics expressing the Cre recombinase driven by the myosin heavy chain 6 (*Myh6*) promoter, which is active since E10.5 [18]. Mutant mice were obtained at the expected Mendelian ratio, were viable and grew to adulthood. This agrees with previous findings indicating that Ezh2 in differentiated CMs is not required for heart development and function, and embryogenesis [11]. Loss of *Ezh2* was confirmed by qPCR, which showed ~65% reduction in mRNA levels in whole ventricles of mutant mice (Fig 1A), consistent with a cardiomyocyte specific loss of *Ezh2*. This loss of *Ezh2* caused a 5-fold reduction in the number of H3K27me3 positive CMs (*p* = 2.26 x 10^−16^), but not in H3K27me3 positive non-CMs (*p* = 0.807), as compared to control hearts. 94 ± 2% control CMs were H3K27me3+, while only 17 ± 1% mutant CMs were H3K27me3+, indicating efficient Ezh2 inactivation (Fig 1B and 1C). This agrees with previous studies showing that Ezh2 is the major H3K27me3 transferase in cardiac myocytes [10].

### The neonatal mouse heart can regenerate, and the regenerative capacity is lost at P7

**Fig 2:**
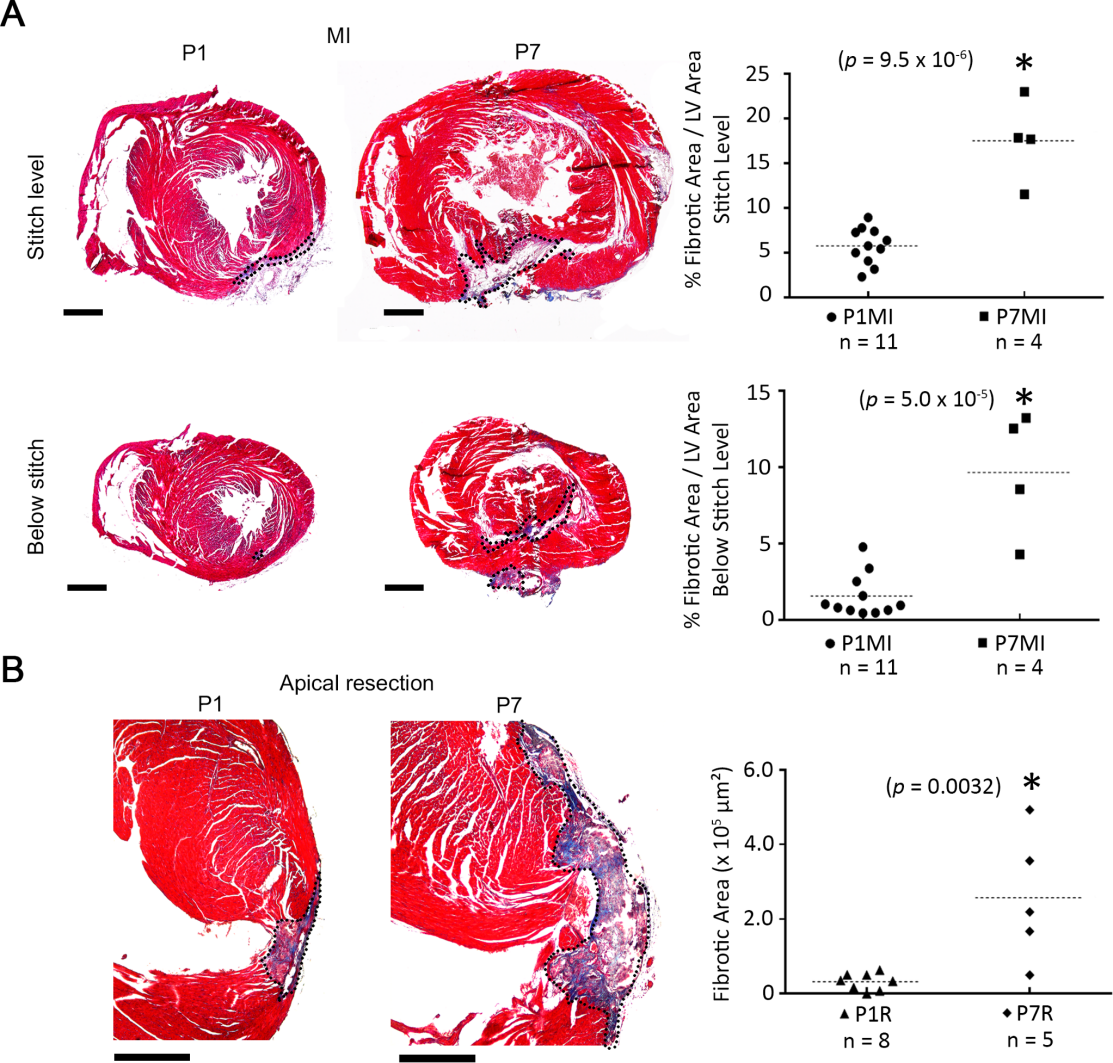
Cardiac regeneration capacity is lost at P7 in wild type mice. (A) Masson’s trichrome-stained cross sections of wild type mouse hearts showing fibrous tissue (blue) at the level of the ligature and below the ligature (stitch) 3 weeks post MI induced at P1 or P7. Plots represent quantification of the fibrous tissue area as percentage area of the left ventricle (LV). (B) Masson’s trichrome-stained sections showing fibrous tissue in the left ventricle of hearts that underwent apical resection at P1 or P7. Hearts were collected 3 weeks post-resection. Plots represent the fibrous tissue area. The scar area is outlined. Dotted lines show the mean in plots. Scale bar = 500 μm. * = p<0.05.

Previous reports from different groups have reported mixed results in the capacity of neonatal mice to regenerate, possibly owing to the degree of injury, or the surgical procedure used to induce myocardium injury [5, 19, 20]. Hence, we first sought to confirm the regenerative capacity of neonatal pups at P1, and that it is lost after 1 week in P7 pups. We induced MI or resected the heart apex at P1 and P7 in wild type pups and assessed the extent of heart regeneration by measuring the fibrotic scar size after 3 weeks. Myocardial infarction at P1 induced significantly less scarring 3 weeks post-resection, compared to MI induced at P7 (*p* = 9.5 x 10^−6^) (Fig 2A). Similarly, ventricular apical resection resulted in a significantly bigger scar 3 weeks post-resection, compared to resection at P1, which resulted in minimal scarring (p = 0.0032) (Fig 2B). This is consistent with previous reports showing that the cardiac regenerative capacity is lost after the first week of life [5, 21]. The scar remaining 3 weeks post-MI in hearts injured at P1 is confined to the region in which the ligature was placed. In contrast, the scar extends beyond the ligature level in hearts injured at P7 (Fig 2A), indicating decreased myocardium regeneration. These results confirm the innate regenerative capacity of the heart at P1, and its loss at P7.

### Ezh2 Is not Required For Innate Neonatal Cardiac Regeneration

**Fig 3:**
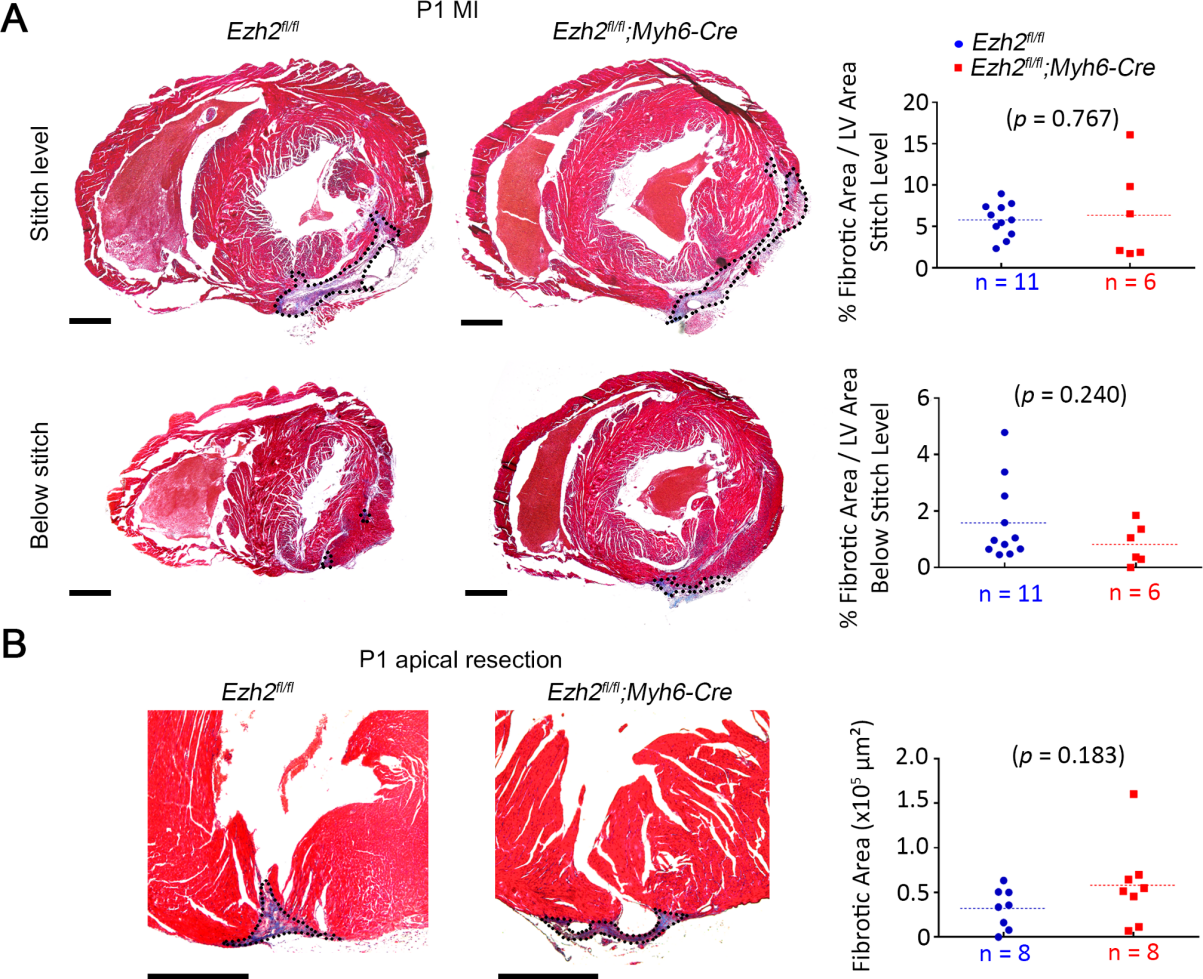
Ezh2 is not required for innate neonatal cardiac regeneration. (A) Masson’s trichrome-stained cross sections showing fibrous tissue at the level of the ligature (stitch) and below the ligature in control (*Ezh2*^*fl/fl*^) and mutant (*Ezh2*^*fl/f*^*;Myh6-Cre*) hearts, 3 weeks post-MI induced at P1. Scar area is outlined. Plots represent quantification of fibrotic scar area as the percentage of the left ventricle (LV) area. (B) Masson’s trichrome-stained sections showing fibrous tissue (blue) at the resection area in control (*Ezh2*^*fl/fl*^) and mutant (*Ezh2*^*fl/fl*^::*Myh6cre+*) hearts 3 weeks post apical resection at P1. Plots represent quantification of the scar area. Scar area is outlined. Dotted lines show the mean in plots. Scale bar = 500μm.

To assess whether *Ezh2* is required for the innate cardiac regenerative response, we induced MI in control wild type (*n* = 11) and *Ezh*2 mutants at P1 (*n* = 6) via ligation of the left anterior descending coronary artery (LAD ligation). The scar size in *Ezh2* mutants was comparable to controls at the suture level, and was almost completely cleared distal from the suture 3 weeks post MI (5.8 ± 0.6% scar area/LV area in mutants, and 6.4 ± 2.4% in controls at suture level; *p* = 0.767) (Fig 3A). Similar results were obtained when inducing myocardial damage by apical resection at P1 (Fibrotic area = 3.2 x 10^4^ ± 7.9 x 10^3^ um^2^ in mutants, and 5.8 x 10^4^ ± 1.7 x 10^4^ um^2^ in controls; *p* = 0.183) (Fig 3B). Thus, CM-specific *Ezh2*-deletion did not affect scar size and did not impede clearance of fibrotic tissue after cardiac injury at P1.

### Ezh2 inactivation does not extend the neonatal regenerative time window

**Fig 4:**
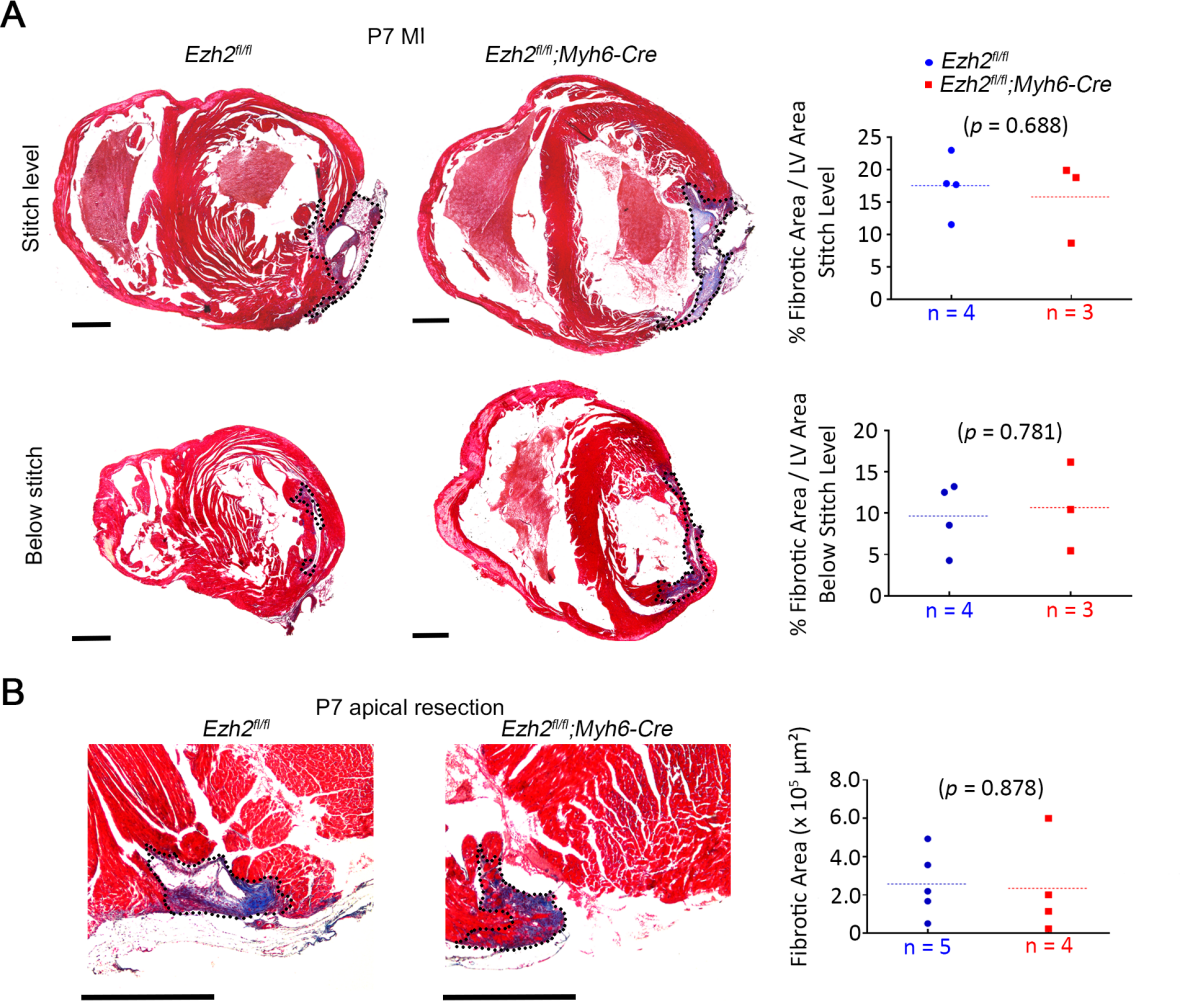
Loss of Ezh2 does not extend the neonatal regenerative time window. (A) Masson’s trichrome-stained sections showing cardiac fibrosis at the level of the ligature (stitch) and below the ligature in control (*Ezh2*^*fl/fl*^) and mutant (*Ezh2*^*fl/fl*^*;Myh6-Cre*) hearts, 3 weeks post-MI induced at P7. Scar area is outlined. Plots represent the quantification of fibrotic scar area as a percentage of the left ventricle area. (B) Masson’s trichrome-stained sections showing fibrous tissue (blue) at the resection area in hearts 3 weeks post-resection induced at P7. Plots represent quantifications of scar area. Scar area is outlined. Scale bar = 500 μm.

To assess whether *Ezh2* deletion prolongs the regenerative capacity past P7, we induced MI in *Ezh2* mutant (*n* = 3) and control (*n* = 4) mice at P7 via LAD ligation. *Ezh2* mutants showed no difference in scar size at the suture level, and both control and mutants cleared the scar (15.8 ± 3.6% scar area/LV area in mutants, and 17.5 ± 2.3% scar area/LV area in controls; *p* = 0.688) (Fig 4A). Similarly, the scar size was comparable between *Ezh2* mutants (*n* = 4) and controls (*n* = 5) three weeks after apical resection induced at P7 (2.3 x 10^5^ ± 1.3 x 10^5^ μm^2^ scar area in mutants, and 2.6 x 10^5^ ± 7.6 x 10^4^ μm^2^ scar area in controls; *p* = 0.878) (Fig 4B).

### Ezh2 deficiency does not affect cell proliferation in the neonatal heart in response to injury

**Fig 5:**
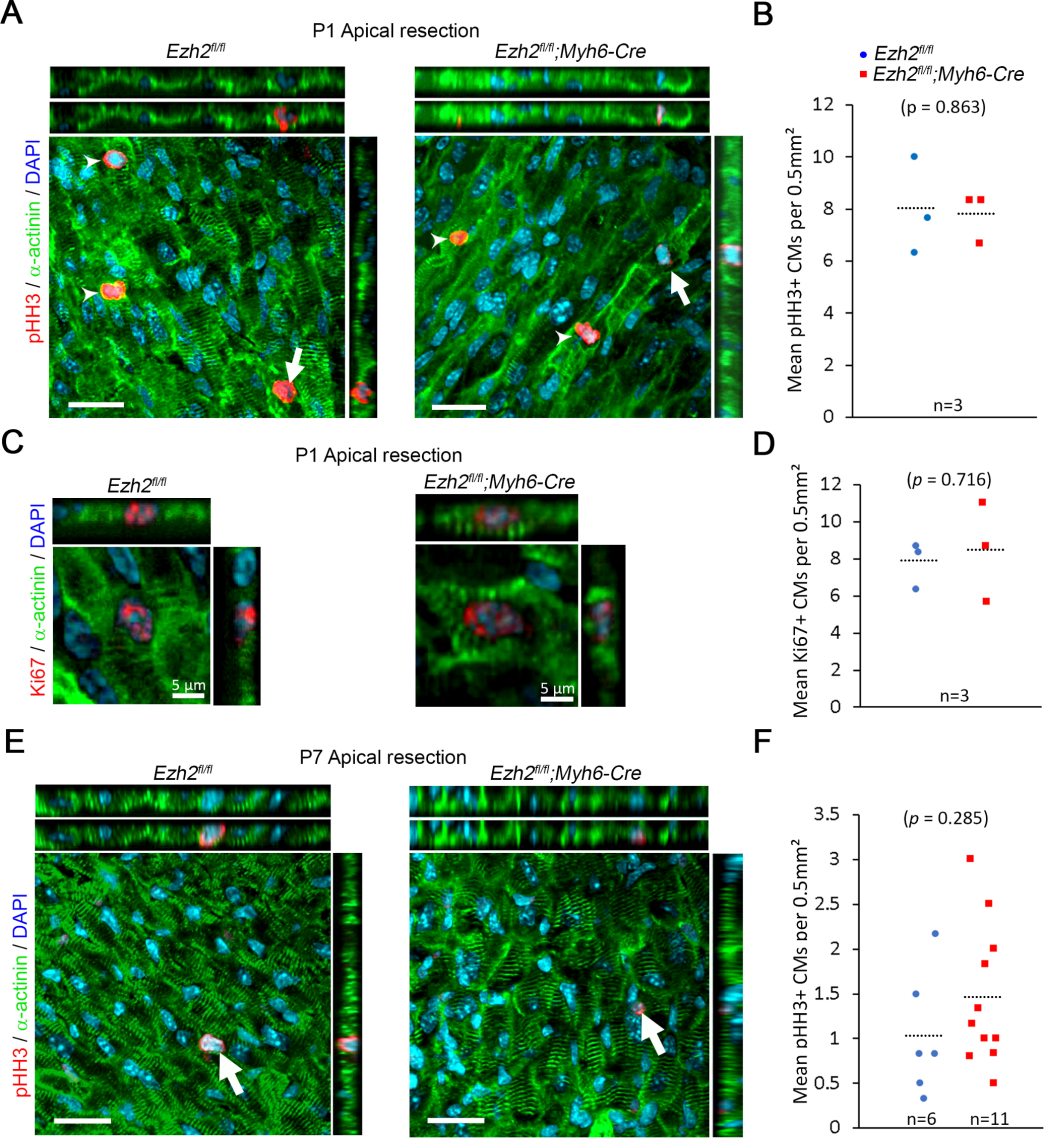
*Ezh2* does not affect cell proliferation in the neonatal heart in response to injury. (A) Immunofluorescence for phosphorylated histone H3 (red) in sections of hearts from control (*Ezh2*^*fl/fl*^) and mutant (*Ezh2*^*fl/fl*^*;Myh6-Cre*) mice 1 week post-resection induced at P1. (B) Quantification of phosphorylated histone H3 (pHH3)-positive cardiomyocytes. (C) Immunofluorescence for Ki67 (red) in sections of hearts from control (*Ezh2*^*fl/fl*^) and mutant (*Ezh2*^*fl/fl*^*;Myh6-Cre*) mice, 1 week post-resection induced at P1. (D) Quantification of Ki76-positive cardiomyocytes. (E) Immunofluorescence for phosphorylated histone 3 (red) in sections of hearts from control (*Ezh2*^*fl/fl*^) and mutant (*Ezh2*^*fl/fl*^*;Myh6-Cre*) mice, 1 week post-resection induced at P7. (F) Quantification of phosphorylated histone H3 (pHH3)-positive cardiomyocytes. α-actinin stained cardiomyocytes (green), and nuclei were counterstained with DAPI (blue). The top and lateral panels show Z-dimension reconstructions. Dotted lines show the mean in plots. Scale bar = 100 μm.

It has been shown that increasing cell proliferation is key for neonatal cardiac regeneration. Hence, we sought to determine if deletion of *Ezh2* had an impact on cell proliferation. We induced myocardium damage by apical resection in P1 and P7 pups to assess CM proliferation at 1 week post-injury by immunofluorescence for phosphorylated histone H3 (pHH3) and Ki67. We found that the number of proliferating CMs was comparable between controls and *Ezh2* mutants in response to injury at P1 or P7 (Fig 5).

## Discussion

The neonatal mammalian heart can activate fetal gene expression programs to promote regeneration, however, this capacity is lost within days after birth [5]. Ezh2 represses fetal gene programs during cardiac development [10]. In this work, we found that deletion of Ezh2 does not affect the extent of fibrosis or cardiomyocyte proliferation after cardiac injury, despite reduction of the histone repressive mark H3K27me3. This suggests that gene repression mediated by the histone methyltransferase activity of Ezh2 does not play a major role in the neonatal heart regenerative response.

Distinct responses to the various methods used to induce cardiac injury in the neonatal mouse [19, 22–24], rose controversy on the capacity of the neonatal mouse to regenerate. We validated two different models of cardiac injury, apical resection and MI. In both models, wild type P1 mice could clear the fibrotic scar, but P7 mice failed to restore lost myocardium. Thus, our work supports previous findings demonstrating the regenerative capacity of the neonatal heart (5,6).

We demonstrated that a function of Ezh2 in CMs is not required for innate cardiac regeneration nor does it extend the regenerative period. Wild type mice can regenerate their hearts following cardiac injury at P1 but not P7. Both mutants and controls showed almost complete recovery from MI injury at P1, excepting the area of the suture [6], but were not able to clear the fibrotic scar after P7 MI. We also tested the role of *Ezh2* in an apical resection model to determine if the response was specific to the type of injury model used. Following apical resection, we found that the scar size between mutants and controls was comparable. It should be noted that we did not observe full regeneration of the resected heart apex at P1. This is likely because our resections are consistent with a previous report showing that resecting a larger portion of the apex results in a persistent small scar [20].

*Ezh2*-inactivation alone is not enough to affect heart regeneration due to compensation by the other H3K27me3 methyltransferase Ezh1. It has recently been demonstrated that Ezh1 can act to compensate for Ezh2 loss of function during regeneration. Indeed, Ezh1 is required for neonatal cardiac regeneration [25]. Our results support the existing model suggesting that Ezh1, but not Ezh2, is the key PRC2 component required for neonatal regeneration.
